# Nitrous Oxide activates layer 5 prefrontal neurons via SK2 channel inhibition for antidepressant effect

**DOI:** 10.21203/rs.3.rs-5141491/v1

**Published:** 2024-11-15

**Authors:** Joseph Cichon, Thomas T. Joseph, Xinguo Lu, Andrzej Z. Wasilczuk, Max B. Kelz, Steven J. Mennerick, Charles F. Zorumski, Peter Nagele

**Affiliations:** 1 Department of Anesthesiology and Critical Care, Perelman School of Medicine, University of Pennsylvania, Philadelphia, PA, USA; 2 Mahoney Institute for Neurosciences, Perelman School of Medicine, University of Pennsylvania, Philadelphia, PA, USA; 3 Department of Psychiatry, Washington University School of Medicine, St. Louis, MO, USA; 4 The Taylor Family Institute for Innovative Psychiatric Research, Washington University School of Medicine; 5 Department of Anesthesia and Critical Care, University of Chicago, Chicago, Illinois

## Abstract

Nitrous oxide (N_2_O) induces rapid and durable antidepressant effects. The cellular and circuit mechanisms mediating this process are not known. Here we find that a single dose of inhaled N_2_O induces rapid and specific activation of layer V (L5) pyramidal neurons in the cingulate cortex of rodents exposed to chronic stress conditions. N_2_O-induced L5 activation rescues a stress-associated hypoactivity state, persists following exposure, and is necessary for its antidepressant-like activity. Although NMDA-receptor antagonism is believed to be a primary mechanism of action for N_2_O, L5 neurons activate even when NMDA-receptor function is attenuated through both pharmacological and genetic approaches. By examining different molecular and circuit targets, we identify N_2_O-induced inhibition of calcium-sensitive potassium (SK2) channels as a key molecular interaction responsible for driving specific L5 activity along with ensuing antidepressant-like effects. These results suggest that N_2_O-induced L5 activation is crucial for its fast antidepressant action and this effect involves novel and specific molecular actions in distinct cortical cell types.

Major depression is a heterogenous condition that diminishes psychosocial functioning and quality of life. Despite advances in understanding pathophysiology and antidepressant mechanisms, up to one-third of patients have failed responses to conventional treatments (1, 2), possibly reflecting different subtypes affecting distinct cell types and neuronal networks (3). Prefrontal cortical (PFC) circuit dysfunction remains most consistently involved with depression severity tied to deficits in neuronal activity, plasticity, and structure (4–7). Both rodent models of chronic stress and depressed human patients show hypoactive PFC networks (4, 8, 9), impaired plasticity (10), and reduced synaptic number and brain volume (11–13). Existing treatments for depression reverse many disease-associated circuit defects but often have slow-onset (3, 14). Psychedelics and anesthetics, such as ketamine and nitrous oxide (N_2_O) (15–17), produce both rapid and sustained antidepressant effects following a single treatment in patients suffering from treatment-resistant depression (TRD). How these novel antidepressants enact their rapid and durable antidepressant effects is poorly understood.

The success of ketamine as a rapid antidepressant reawakened interest in N_2_O as a possible antidepressant (18, 19). Two randomized controlled, early-phase clinical trials of N_2_O (25–50%) demonstrated promising results where TRD patients experienced rapid and sustained symptomatic relief, mirroring the rapid antidepressant effects of ketamine (17, 18). Like ketamine, N_2_O’s molecular mechanism on neuronal function is thought to involve NMDA-receptor (NMDA-R) antagonism (20, 21), but this has never been directly tested *in vivo*. It remains unclear how N_2_O could drive rapid and durable therapeutic effects despite fast elimination from the brain (~5 min) and no metabolites (22). Here, we investigated the cellular and circuit basis for N_2_O’s antidepressant effect by imaging PFC microcircuits before and after N_2_O treatment.

## N_2_O’s antidepressant-like response arises from rapid activation of L5 pyramidal neurons

**Fig. 1.**
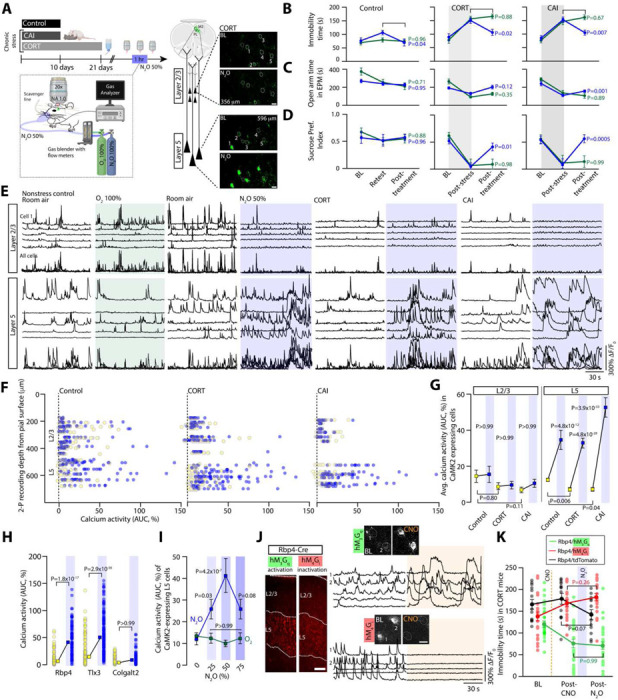
N_2_O induces rapid and specific activation of L5 pyramidal neurons to drive antidepressant-like response. **A**, Left, chronic stress was induced with either corticosterone in tap water (CORT) or exposure to screened aggressive male CD-1 mouse (chronic aggressor interactions or CAI). CORT, CAI, and control (exposed to daily handling) mice were subjected to two-photon calcium imaging in the Cg1 (imaging location denoted by green highlighted region with dashed line to cell types) before, during, and after nitrous oxide (N_2_O) exposure. N_2_O was blended, delivered, and monitored under the microscope at 50% for 1 hr. Right, CaMK2-expressing excitatory neurons located in layer 2/3 (L2/3) and layer 5 (L5) in CORT mice. Top (room air) and bottom (N_2_O) images show 5 cells from each layer with their fluorescent transients from a 2 min time-series movie collapsed into a single image. Scale bar, 20 μm. **B-D**, Chronic stress (CORT, N_2_O *n* = 16, O_2_ *n* = 12; CAI, N_2_O *n* = 11, O_2_ *n* = 10) increased the time spent immobile in tail suspension test (TST) (**B**, avg. immobility time was 153 ± 7 seconds in CORT and 149 ± 8 seconds in CAI vs. 86 ± 7 seconds in controls; Kruskal-Wallis (33): *P* = 5.6 × 10^−8^ followed by Dunn’s multiple comparisons, control vs. CORT: *P* = 4.1 × 10^−7^, control vs. CAI: *P* = 6.8 × 10^−6^), decreased exploration of open arms in an elevated plus maze (EPM) (**C**, avg. open arm time was 113 ± 11 seconds in CORT and 115 ± 16 seconds in CAI vs. 238 ± 19 seconds in control mice; Kruskal-Wallis (31): *P* = 1.6 × 10^−7^ followed by Dunn’s multiple comparisons, control vs. CORT: *P* = 1.3 × 10^−6^, control vs. CAI: *P* = 1.0 × 10^−5^), and reduced sucrose preference index (SPI) (**D**, avg. SPI was 0.06 ± 0.1 in CORT and 0.1 ± 0.1 in CAI vs. 0.5 ± 0.1 in control mice; Kruskal-Wallis (22): *P* = 1.6 × 10^−7^ followed by Dunn’s multiple comparisons, control vs. CORT: *P* = 2.5 × 10^−5^, control vs. CAI: *P* = 8.7 × 10^−4^), as compared to control mice (N_2_O *n* = 14, O_2_ *n* = 14). N_2_O, but not O_2_, therapy rapidly reversed the effects of chronic stress (2-way ANOVA time x treatment: TST CORT, F_(2, 78)_ = 8.1, *P* = 8.6 × 10^−4^, TST CAI, F_(2, 38)_ = 7.4, *P* = 0.002; EPM CORT, F_(2, 78)_ = 6.5, *P* = 0.002, EPM CAI, F_(2, 38)_ = 3.0, *P* = 0.06; SPI CORT, F_(2, 52)_ = 4.0, *P* = 0.02, SPI CAI, F_(2, 57)_ = 7.6, *P* = 0.001). Post treatment comparisons (N_2_O, blue; O_2_, green) are shown in panel. **E**, Representative GCaMP6 traces of the spontaneous activity of individual neurons from L2/3 and L5, shown in **A**, under wakefulness followed by oxygen (left, green) or N_2_O (right, blue) in control and chronically stressed mice. N_2_O induced the rapid recruitment of L5 neurons as compared to L2/3 across all conditions. **F**, Individual neuronal responses (circles) under room air (yellow) and N_2_O (blue) from all recording regions across Cg1 from control (left) and chronically stressed (middle/CORT and right/CAI) mice. Oxygen plot in Fig. S4A. **G**, L2/3 and L5 population response (colored squares) under room air and N_2_O across control (L2/3: *n* = 121; L5: *n* = 102 from 8 mice) and CORT (L2/3: *n* = 96; L5: *n* = 122 from 9 mice)/CAI mice (L2/3: *n* = 61; L5: *n* = 98 from 4 mice). N_2_O drove the rapid L5 activation across stressed and control mice (Kruskal-Wallis (390): *P* = 5.3 × 10^−77^ followed by Dunn’s multiple comparisons, control: *P* = 4.8 × 10^−12^, CORT: *P* = 4.8 × 10^−28^, CAI: *P* = 3.9 × 10^−33^) as opposed to L2/3 (Kruskal-Wallis: control/CORT/CAI: *P* > 0.99). L5 neurons from chronically stressed mice displayed a hypoactivity state relative to control mice (from prior Dunn’s test: CORT, *P* = 0.006; CAI, *P* = 0.04). L2/3 activity was also reduced but not significant (CORT, *P* = 0.80; CAI, *P* = 0.11). **H**, L5 calcium responses under room air and N_2_O across different genetically defined L5 neuronal subtypes (Kruskal-Wallis (404): *P* = 4.4 × 10^−85^ followed by Dunn’s multiple comparisons, Rbp4 (*n* = 89 from 3 mice): *P* = 1.8 × 10^−17^, Tlx3 (*n* = 196 from 4 mice): *P* = 2.9 × 10^−36^; Colgalt2 (*n* = 149 from 4 mice): *P* > 0.99). **I**, CaMK2-expressing L5 neuronal responses at different N_2_O concentrations (0, 25, 50, 75% mixed with O_2_). L5 activating effect observed at 25% with peak effect at 50% (Two-way ANOVA with Bonferroni’s comparisons: 25%, *P* = 0.03; 50%, *P* = 4.2 × 10^−7^; 75%, *P* = 0.08). **J**, Left, coronal sections of Cg1 from Rbp4-Cre mouse expressing either Cre-dependent chemogenetic variant (tagged with mCherry) hM_3_G_q_ (green) or hM_4_G_i_ (red; Scale bar 100 μm). Right, representative GCaMP6 traces (bottom) and two-photon images (top) of individual L5 neurons recorded during room air and following CNO injection (orange shared area). Scale bar, 20 μm. **K**, CNO-induced Rbp4-L5 neuronal inactivation with hM_4_G_i_ bilaterally in Cg1 (*n* = 22) blocked N_2_O’s effect on TST whereas CNO-induced Rbp4-L5 activation with hM_3_G_q_ (*n* = 14) masked N_2_O’s effect (two-way ANOVA time x treatment F_(4, 166)_ = 19.8, *P* = 1.9 × 10^−12^ post hoc Sidak’s comparisons shown in panel following N_2_O exposure. Representative images and traces carried out on at least three animals per group. Error bars show s.e.m.

Chronic stress is one important risk factor for depression (23). In animal models, chronic stress recapitulates key features of the depressed brain, including maladaptive changes in neuronal structure, function, and behaviors (24). In this work, we chronically stressed mice with two different strategies, 1) chronic corticosterone (referred to as CORT) in drinking water (25, 26) and 2) chronic exposure of a male C57BL/6 mouse to a male CD-1 aggressor mouse (termed chronic aggressor interactions or CAI)(Fig. 1A, fig. S1A-C, Supplemental Movie 1) (27, 28). Following chronic stress, animals displayed an anxiodepressive-like state as evidenced by prolonged immobility time in tail suspension test (TST)), decreased preference for sucrose in sucrose preference test, and reduced activity in open arms in the elevated plus maze as compared to controls exposed to daily handling (Fig. 1B–D). To determine if N_2_O exhibits rapid antidepressant-like effects in rodents exposed to chronic stress, we administered inhaled N_2_O (50%) or O_2_ (control; 100%) for 1 hr to head-restrained mice via a nose cone (Fig. 1A, fig. S1D). We chose a 50% N_2_O dose instead of 25% because of the open-circuit delivery method in rodent studies, unlike the semi-closed-circuit approach commonly used in human trials. We found that N_2_O drove the rapid (within 1 hr from treatment) reversal of chronic stress associated behaviors in both CORT and CAI mice compared to O_2_-treated controls (Fig. 1B–D). In a separate cohort of mice tracked only in TST we observed a sustained decrease in immobility time at 24 hr post treatment in both CORT and CAI mice but not in controls (fig. S1F). Although it is difficult to assess drug-induced behavioral effects in head-restrained mice, unrestrained mice in a closed chamber exposed to 50% N_2_O showed increased movement and exploration rather than signs of sedation (fig. S2).

Dysfunction of the medial PFC is a hallmark feature of human depression (6) and preclinical models of chronic stress (29, 30). To determine if chronic stress produces a similar effect in rodent brain, we recorded the spontaneous activity of excitatory neurons across the cortical column in the cingulate cortex (Cg1), which is a supplemental motor area in rodents, by using two-photon (2-P) calcium imaging (Fig. 1A, right). Adeno-associated virus (AAV) encoding the calcium indicator GCaMP6f under the Calcium/Calmodulin dependent protein kinase II (CaMK2) promoter drove stable sensor expression specific to pyramidal neurons of superficial, *i.e*. layer (L) 2/3, and deep, L5, layers in this location (fig. S3C). Deep neurons of chronically stressed mice (CORT and CAI) showed a hypoactivity state in L5 as compared to controls (Fig. 1E, G), a finding consistent with this region reconfiguring to chronic stress (8). Thus, in agreement with previous reports (29), chronic stress in adult rodents induces both behavioral and neurophysiological changes consistent with a depression-like state.

Considering that N_2_O is believed to reduce both pre- and postsynaptic neuronal activity as a sedative-hypnotic with neuroprotective properties (20, 31), it was unclear how N_2_O would produce its rapid antidepressant effect in a stress-induced hypoactive cortical network. Here, inhaled N_2_O (50%) induced rapid activation of deep pyramidal neurons (Avg. cell location ± std. error of mean, control: 573 ± 17 μm, CORT: 623 ± 14 μm, CAI: 619 ± 16 μm) in both chronically stressed and control mice as compared to both superficially located L2/3 neurons (control: 315 ± 20 μm, CORT: 297 ± 22 μm, CAI: 272 ± 24 μm) and O_2_ controls from the same region (Fig. 1E–G, fig. S4A, D, Supplemental Movie 2). Comparisons were made after 15 min of N_2_O exposure to ensure steady state gas concentration. Considering Cg1’s anatomical location and cytoarchitecture, we proposed the deep pyramidal neurons recruited by N_2_O are likely within L5. L5 identity was further supported by calcium imaging in specific L5 transgenic Cre lines, where N_2_O recruited Rbp4 and Tlx3 expressing cells, but not Colgalt2 (Fig. 1H) (32). Furthermore, calcium imaging of CaMK2-expressing cells at a depth of >500–600 μm from the pial surface in secondary motor cortex (fig. S4B) and primary somatosensory cortex (fig. S4B-C) also revealed putative L5 neuronal activation as compared to L2/3. Therefore, we refer to this N_2_O-recruited deep neuronal population as L5 hereon.

Within a local imaging field of view, N_2_O-active L5 cells generated large asynchronous calcium transients, resembling a form of burst-like firing (fig. S4E-G) (33). Given the sustained nature of these transients, we show calcium activity as area under the curve (AUC) of the **Δ**F/F_0_ trace for an individual neuron, reflecting changes in both frequency and amplitude (fig. S4H). A dose-response curve of CaMK2-expressing L5 population in Cg1 revealed a peak activation effect at 50% as opposed to 25 or 75% N_2_O (Fig. 1I, fig. S1E). Electroencephalogram (EEG) recordings over the same N_2_O concentration steps revealed higher frequency oscillations at 50% as compared to 25 or 75% (fig. S5). These results suggest that at doses found to have antidepressant effects in human patients suffering from TRD (17, 18), N_2_O inhalation drives a rapid activation of the same class of neurons most profoundly affected by chronic stress in rodents (Fig. 1G) (29).

Because N_2_O activates L5 from its baseline hypoactive state in chronically stressed mice, we wondered if this activation plays a pivotal role in N_2_O’s antidepressant response. To specifically increase L5 activity *in vivo* (in absence of N_2_O), we specifically transfected Rbp4 Cre-expressing mice with an AAV encoding Cre-dependent hM_3_D(G_q_) designer receptor exclusively activated by designer drug (DREADD) receptor in Cg1 bilaterally (Fig. 1J, fig. S6A). The binding of the ligand clozapine *N*-oxide (CNO) to hM_3_D(G_q_) receptors activates G_q_-coupled signaling, leading to membrane depolarization and increased firing of target cells through multiple mechanisms (34). CNO delivered by i.p. injection to mice expressing hM_3_D(G_q_) specifically in L5 cells induced a ~2–3-fold increase in spontaneous calcium activity (when assessed at 1 and 3 hrs later) and a rapid antidepressant-like effect detected within 1 hr that lasted at least for 1 day (Fig. 1J, fig. S6B-D). This behavioral effect induced by hM_3_D(G_q_) in L5 masked N_2_O’s effect when CNO was delivered prior to N_2_O (Fig. 1K). Conversely, L5 inactivation by DREADD-hM_4_D(G_i_) acutely blocked N_2_O’s ability to recruit L5 and its accompanying antidepressant effect when CNO was delivered prior to N_2_O (Fig. 1J–K, fig. S6B). These results suggest that a single N_2_O treatment induces rapid and specific activation of L5 neurons in Cg1 to rescue chronic stress-associated hypoactivity and that recruitment of these cells is required for N_2_O’s ensuing antidepressant-like effect.

## N_2_O-induced rescue of stress associated L5 hypoactivity persists following drug elimination

**Fig. 2.**
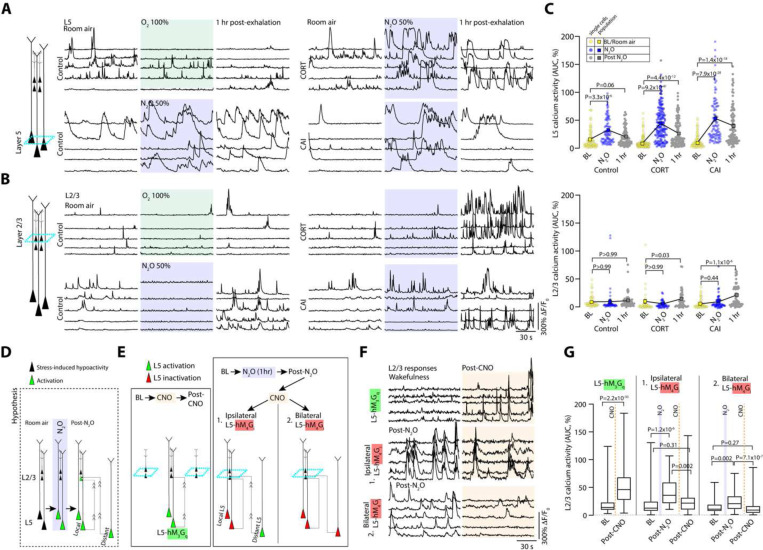
Persistent L5 activity following N_2_O elimination contributes to L2/3 recruitment. **A-B**, The same individual L5 (**A**) and L2/3 (**B**) neuronal responses under room air (left), O_2_, or N_2_O (middle), 1 hr after O_2_ or N_2_O (right) in control, CORT-treated and CAI mice . Note the persistent activity of L5 neurons and recruitment of L2/3 neurons following N_2_O . **C**, Summary of individual (circles) and population (colored squares connected by black line) neuronal responses of both L5 and L2/3 neurons across wakefulness, N_2_O exposure, and following N_2_O exhalation in control (L2/3: *n* = 52; L5: *n* = 88 from 3 mice) and CORT (L2/3: *n* = 43; L5: *n* = 163 from 5 mice)/CAI (L2/3: *n* = 61; L5: *n* = 97 from 4 mice) mice. Cohort different from Fig. 1). Persistent activation of L5 was observed at 1 hr following N_2_O exposure in all groups (Kruskal-Wallis (559): *P* = 5.5 × 10^−108^ followed by Dunn’s multiple comparisons, control, *P* = 0.06; CORT, *P* = 4.4 × 10^−12^; CAI, *P* = 1.4 × 10^−18^) while significant L2/3 activation was observed in only CORT-treated and CAI groups at 1 hr following N_2_O exposure (prior Dunn’s test: control, *P* > 0.99; CORT, *P* = 0.03; CAI, *P* = 1.1 × 10^−6^). **D**, Schematic illustrating that hypoactive L5 neurons (black cells) become rapidly activated by N_2_O treatment (green cells). Following N_2_O exhalation, persistent L5 activity, from local or distant regions (green activated cells), may recruit L2/3 (superficial green cells receiving projections). **E**, Experimental design to test whether 1) direct local chemogenetic L5 activation results in recruitment of local L2/3 neurons (left), 2) Post-N_2_O recruitment of L2/3 is dependent upon persistent local and distant L5 activity. **F**, Individual L2/3 neurons responses corresponding to **E**. Top, L2/3 responses before and after CNO-induced activation of L5 neurons expressing hM_3_G_q_ (*n* = 161 from 5 mice). Middle and bottom traces, L2/3 responses following either ipsilateral (*n* = 42 from 2 mice) or bilateral (*n* = 77 from 3 mice) hM_4_G_i_-mediated L5 inhibition respectively. **G**, Summary of individual L2/3 responses from **E**. CNO-induced activation of L5-hM_3_G_q_ drove superficial L2/3 activity (Wilcoxon matched-pairs signed rank test: *P* = 2.2 × 10^−30^). Either ipsilateral (Kruskal-Wallis (26.8): *P* = 1.5 × 10^−6^ followed by Dunn’s multiple comparisons, post-N_2_O: *P* = 1.2 × 10^−6^; post-CNO: *P* = 0.31) or bilateral (Kruskal-Wallis (26.9): *P* = 1.4 × 10^−6^ followed by Dunn’s multiple comparisons, post-N_2_O: *P* = 0.002; post-CNO: *P* = 0.27) CNO-induced L5-hM_4_G_i_ inhibition reduced post-N_2_O-induced L2/3 activity to baseline wakefulness. Error bars show s.e.m.

N_2_O is rapidly cleared from the brain/body within minutes (via expiration) without active metabolites (35). To determine if N_2_O’s acute L5 activation contributes to its lasting antidepressant-like effect, we followed the same L5 populations following N_2_O exhalation. To our surprise, we found persistent L5 activity in both chronically stressed and control mice 1 hr following N_2_O exposure but not in O_2_ treated mice (Fig. 2A, C, fig. S7A). L5 responses persisted for at least 3 hrs in chronically stressed mice whereas control mice showed a normalization to baseline activity patterns recorded under room air (fig. S7B). In some cases where L5 was tracked over 24 hours, we found evidence of persistent activity (fig. S7D). At these follow-up time points, superficial L2/3 cells, previously weakly active or not active, were now found to be spontaneously active (Fig. 2B–C). Newly active L2/3 activity also persisted for hours (Fig. 2B–C, fig. S7D).

Given that L5 was recruited during N_2_O exposure and both L5 and L2/3 have persistent spontaneous activity post-treatment, we suspected that N_2_O-L5 activation contributes to the recruitment and sustained activation of local L2/3 neurons in Cg1 (Fig. 2D). To determine if local L5 neuronal activity can drive local L2/3 activation, L5-specific Rbp4-Cre mice were transfected with AAVs encoding Cre-dependent hM_3_D(G_q_) and synapsin-GCaMP6f for DREADD-induced L5 activation while recording L2/3 calcium responses respectively (Fig. 2E, left schematic). CNO-induced L5 activation drove L2/3 activation (2.5-fold increase in spontaneous activity) 20–30 min following CNO injection (Fig. 2F–G). To ascertain if post-N_2_O exposure L5 activity drives local L2/3 activity in chronically stressed mice, CORT-treated Rbp4 mice expressing hM_4_D(G_i_) (unilateral expression) were exposed to N_2_O to drive L5 activation and persistent activity. L5 neurons were then chemogenetically inactivated by hM_4_G_i_, meanwhile recording ipsilateral L2/3 neuronal activity (Fig. 2E, right schematic). Following CNO injection (imaging 20–30 min after CNO), post N_2_O-induced L2/3 activity was significantly reduced by L5 inactivation (Fig. 2F–G). We suspected the residual L2/3 activity (~50% increase over baseline) following local L5 silencing could be explained by long-range L5 projections (both ipsilateral and contralateral) into Cg1 (Fig. 2E). In agreement with this hypothesis, bilateral inhibition of Cg1 L5 neurons post-N_2_O further reduced spontaneous L2/3 activity (~18% decrease from baseline) (Fig. 2F–G). Furthermore, bilateral inhibition of both L2/3 and L5 activity post-N_2_O via CNO-induced hM_4_G_i_ signaling promoted the reversal N_2_O-induced antidepressant effect (fig. S7C). These experiments demonstrate that N_2_O exposure drives the rapid and persistent activity of L5 neurons. Following N_2_O elimination, L5 neurons contribute to the recruitment of excitatory neurons in interconnected circuits, which contributes N_2_O’s antidepressant-like response.

## Reduced NMDA-R function does not block N_2_O-induced L5 activation

**Fig. 3.**
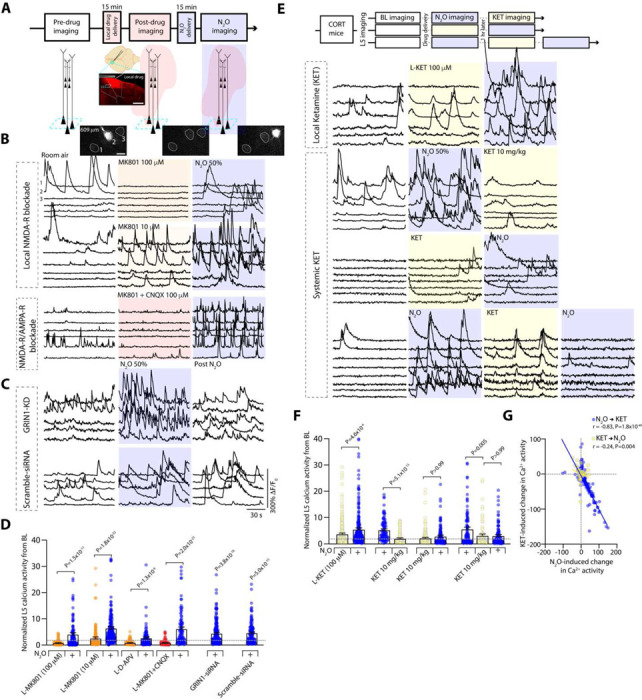
Reduced NMDA-receptor activity does not impair N_2_O-induced L5 activation *in vivo*. **A**, Top, timeline of L5 imaging (teal box) before, following local (L-) drug application (red cloud), and during N_2_O exposure (blue shaded region). Middle, cartoon and coronal section of Cg1 region after pressure application of MK801 mixed with Rhodamine 6G. Scale bar, 500 μm. Bottom, Representative two-photon images of peak GCaMP6 signal from Cg1 L5 neurons under room air conditions, L-MK801, N_2_O. **C**, Representative GCaMP6 traces of L5 neurons under room air conditions, L-MK801 (top – high concentration/100 μM; *n* = 103 from 3 mice; middle – low concentration/10 μM; *n* = 202 from 4 mice), or L-MK801 + CNQX (lower traces; each drug at 100 μM mixed; *n* = 96 from 3 mice) followed by N_2_O exposure. **C**, Representative GCaMP6 traces of L5 neurons from mice infected with siRNA specific to GRIN1 (top; *n* = 165 from 4 mice) and scramble-siRNA (bottom; *n* = 116 from 4 mice) under room air, N_2_O, following N_2_O. **D**, Summary of individual L5 responses (from **B-C**) normalized to its baseline captured under room air conditions (Kruskal-Wallis (513): *P* = 4.3 × 10^−103^ followed by Dunn’s multiple comparisons, L-MK801 100 μM, *P* = 1.5 × 10^−11^; L-MK801 10 μM, *P* = 1.8 × 10^−22^; L-APV, *P* = x 10^−6^; L-MK801 + CNQX, *P* = 2.0 × 10^−23^; GRIN1-siRNA, *P* = 3.8 × 10^−18^; Scramble siRNA, *P* = 5.0 × 10^−15^). **E**, L5 imaging of CORT mice exposed different drug sequences. Top, room air, L-ketamine (100 μM; *n* = 243 from 4 mice), followed by N_2_O. Middle 2 traces, room air, N_2_O exposure, followed by systemic ketamine (10 mg/kg i.p. injection; *n* = 88 neurons from 3 mice) and its reverse order (ketamine then N_2_O; *n* = 140 neurons from 4 mice). Bottom, room air → N_2_O → systemic ketamine → N_2_O re-exposure (*n* = 104 neurons from 3 mice). **F**, L5 responses from **E** (Kruskal-Wallis (135): *P* = 2.5 × 10^−25^ followed by Dunn’s multiple comparisons, L-ketamine → N_2_O, *P* = 4.6 × 10^−4^; N_2_O → ketamine, *P* = 5.1 × 10^−13^; ketamine → N_2_O, *P* > 0.99; N_2_O → ketamine → N_2_O, *P* = 0.005; N_2_O → ketamine → N_2_O, *P* > 0.99). **G**, N_2_O-induced activity was negatively correlated with ketamine-induced activity in L5 neurons (Pearson correlation: N_2_O → ketamine, *r* = −0.83, *P* = 1.8 × 10^−49^; ketamine → N_2_O, *r* = −0.24, *P* = 0.004). Error bars show s.e.m.

N_2_O-induced L5 rapid and persistent activity is seemingly at odds with N_2_O’s known molecular mechanism as an NMDA-R antagonist as NMDA-R block would likely attenuate neuronal activity (36–38). To determine if NMDA-R activity is required for N_2_O-induced L5 rapid and persistent activity, we recorded L5 responses before and after local application NMDA-R antagonists (both D-APV and MK801) delivered to Cg1 through a small bone hole lateral to the imaging region (39, 40), and once again in the presence of N_2_O (Fig. 3A). Consistent with the expected effects of potent NMDA-R antagonists, both D-APV and MK801 (100 μM, 1 μL) suppressed spontaneous L5 calcium activity. N_2_O, however, retained its ability to recruit L5 activity even in the presence of NMDA-R blockers (Fig. 3B, D, fig. S8A). Lower concentrations of MK801 (10 μM, 1 μL) yielded a similar N_2_O-induced effect despite increasing baseline L5 activity (Fig. 3B, D). Furthermore, L5 activity even persisted following local co-application of potent excitatory synaptic blockers - MK801 and AMPA receptor blocker CNQX (each 100 μM, total volume 1 μL; Fig. 3B, D). Thus, in the presence of either NMDA-R blockers or excitatory synaptic blockers, N_2_O retains its ability to rapidly activate L5.

When L5 neurons were followed post-N_2_O exposure, NMDA-R blockers significantly reduced N_2_O’s effect on persistent L5 activity (fig. S8A). Similarly, in mice first treated with N_2_O and then exposed to local NMDA-R blocker, N_2_O-induced L5 persistent activity was significantly dampened (fig. S8A-B). Therefore, L5 activation and persistent activity could be manifested by two distinct mechanisms: 1) an unknown, NMDA-R-independent activation mechanism, 2) persistent activity maintained by NMDA-R-dependent activity. In support of this claim, N_2_O inhalation drove L5 activation in the presence of NMDA-R subunit 1 (NR1) expression knockdown via siRNA targeted to GRIN1, but with a significant impairment in persistent activity at 1 hr (Fig. 3C–D, fig. S8C). Scrambled siRNA showed no impairments in L5 activity during or post N_2_O exposure (Fig. 3C–D). Thus, contrary to its proposed mechanism of action through NMDA-R antagonism, our findings show that N_2_O rapidly activates L5 neurons even when NMDA-R function is diminished.

Similar to N₂O, ketamine is believed to exert its rapid and long-lasting antidepressant effects primarily through NMDA receptor antagonism (15, 16). Given that N₂O-induced activation of L5 neurons occurs even with reduced NMDA receptor function, we hypothesized that ketamine and N₂O would modulate the spontaneous activity of local Cg1 L5 neurons in distinct ways. To this end, we followed L5 populations through different iterations of N_2_O and subhypnotic ketamine exposures and found L5 neurons displayed opposing modulation by the two drugs (Fig 3E–G). While N_2_O drove the stronger L5 response between the two treatments, ketamine-activated L5 neurons showed little overlap with N_2_O-activated cohort (Fig 3G). Local application of ketamine (100 μM, 1 μL) failed to block or recapitulate N_2_O-induced L5 activation (Fig. 3E–F). When systemic ketamine was delivered before or in between two N_2_O exposures, ketamine inhibited the succeeding N_2_O-induced L5 activation (Fig 3E–F). Therefore, N_2_O and ketamine have highly divergent modulations of L5 activity. This implies both drugs likely engage different cellular and circuit mechanisms to achieve their acute antidepressant effects.

**Fig. 4.**
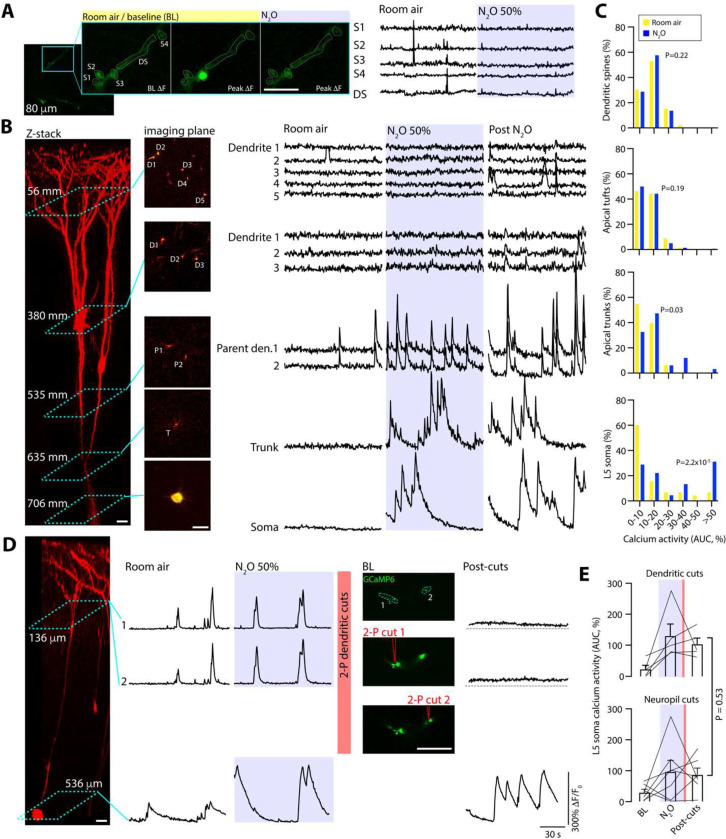
N_2_O fails to recruit synapses and dendrites to drive L5 activity. **A**, Representative two-photon GCaMP6 image of L5 apical dendritic segment (DS; teal box) containing 4 spines (S1, S2, S3, S4) in 2-D imaging plane. Spontaneous spine activity (middle image showing peak signal of single spine activation) is occasionally observed in min recordings under room air conditions. N_2_O fails to recruit spine activity (*n* = 66 spines from 9 mice, Wilcoxon matched-pairs signed rank test: *P* = 0.22). Scale bar, 5 μm. **B**, Left, two-photon z-stack of an individual L5 neuron coexpressing GCaMP6 and tdTomato. Middle, multiple 2-D imaging planes across L5 neuron from apical tuft dendrites to soma corresponding to the teal boxes located on z-stack. ROI labeled D for apical dendrite, P for parent dendrite, T for trunk. Right, GCaMP6 traces for ROIs under room air, N_2_O inhalation, and following N_2_O exhalation. N_2_O fails to recruit superficial dendrites during treatment. Dendritic activity is found deep, in close proximity to soma. Scale bar, 20 μm. C, Summary of calcium responses from across different L5 dendritic compartments (dendritic segments: *n* = 170 from 21 mice, Wilcoxon matched-pairs signed rank test: *P* = 0.19; deep dendrites/trunks: *n* = 34 from 14 mice, Wilcoxon matched-pairs signed rank test: *P* = 0.03) and soma (*n* = 47 from 19 mice, Wilcoxon matched-pairs signed rank test: *P* = 2.2 × 10^−5^). **D**, Left, Z-stack of L5 neuron coexpressing GCaMP6 and tdTomato subject to dendritomies by 2-photon laser pulses. Right, GCaMP6 traces of dendritic ROIs and soma under wakefulness, N_2_O, and following dendritomies. L5 soma and dendrites were activated by N_2_O as compared to wakefulness. GCaMP images of two parents dendrites corresponding to teal box in Z-stack (ROI 1 and 2 at 136 μm) are targeted and sequentially cut using laser pulses resulting in a baseline fluorescence bump coupled with the elimination of transients. L5 activity persists following dendritomies despite loss of dendritic activity. Scale bar, 20 μm. **E**, Top, Summary of individual L5 calcium activity following N_2_O exposure and apical dendritomies (*n* = 5 L5 neurons from 5 mice). Bottom, L5 neurons under same conditions exposed to focal two-photon laser pulses directed at neuropil (control; *n* = 7 L5 neurons from 7 mice). L5 responses following neuropil pulses were not significantly different than those directed at dendrites (Mann Whitney rank sum (13): *P* = 0.53). Error bars show s.e.m.

N_2_O-induced L5 recruitment in the presence of synaptic blockers suggests a synaptic-independent mechanism. To specifically address this hypothesis, we performed dendritic imaging across individually labelled L5 neurons (different cohort from mice in Fig. 1–3). Using a sparse AAV labelling approach, individual L5 neurons expressing both GCaMP6 and tdTomato (structural marker) can be mapped within an imaging window from apical dendritic tree down to L5 soma (Fig. 4B). In dendritic segments confined to layer 1 (63 ± 8.3 μm), N_2_O did not significantly increase the spontaneous activity of postsynaptic dendritic spines nor the generation of dendritic branch calcium events (Fig. 4A–C, fig. S9A). Deeper imaging of dendritic branch points (tuft: 100 ± 7.6 μm) also revealed no significant elevation in spontaneous calcium events comparable to baseline (Fig 4B–C). L2/3 neurons were occasionally labelled with this technique (316 ± 21 μm) were not recruited by N_2_O (fig. S9B). Imaging at the location of L5 soma (627 ± 15 μm) and trunks (333 ± 31 μm) demonstrated robust N_2_O-induced activation (Fig. 4B–C, fig. S9A).

While high-resolution imaging of dendritic segments captures a small portion of the L5 neuron’s apical tuft at a given time (Fig. 4A–B), it is conceivable that other dendritic branches are active and contributing top-down inputs that were missed. To address this possibility, we performed two-photon laser directed apical tuft dendritic cuts (41), or dendritomies, from L5 cells during N_2_O administration and recorded the impact on L5 responses. N_2_O still drove L5 activation despite apical dendrites being physically separated from their soma (Fig. 4D–E, fig. S9C). Furthermore, in an *in vitro* low-density primary cortical neuronal culture (free of network variables), where synaptic connectivity is reduced (no detectable spontaneous calcium activity prior to N_2_O), N_2_O-bubbled bath solution induced the rapid activation of a subset of putative pyramidal cortical neurons (fig. S10). Therefore, N_2_O-induced L5 activation arises independently of NMDA-R activity and synaptic inputs and likely engages a novel, unknown somatic activation mechanism. Nevertheless, the persistent L5 activity observed following N_2_O exposure requires NMDA-R activity.

## N_2_O-induced disinhibition of GABAergic interneurons promotes L5 activity and antidepressant-like effects

The absence of enhanced synaptic inputs or organized dendritic activity suggests N_2_O is driving rapid changes in L5 excitability through a novel somatic mechanism: either a molecular interaction, circuit reconfiguration, or a combination of the two. To this end, we evaluated several candidate channels and receptors that could hypothetically contribute to changes in L5 excitability by imaging L5 responses before and after local delivery of specific channel blockers followed by N_2_O. Functional attenuation of voltage-gated sodium channels, voltage-gated calcium channels, serotonin reuptake transporters, mu opioid receptors, and channels controlling the intracellular release of calcium stores (both IP3 and ryanodine) failed to block N_2_O’s rapid L5 activation (fig. S11A-B). Despite N_2_O-induced L5 recruitment under these conditions, we did observe predicted changes in L5 calcium signal strength and duration imposed by the local drug indicating adequate diffusion to L5 (fig. S11A-B).

Next, we evaluated whether GABA receptor neuromodulation could regulate N_2_O-induced activity. By taking advantage of the GABAergic volatile anesthetic gas isoflurane, which is often coadministrated with N_2_O in clinical practice, we found that subhypnotic isoflurane concentrations, both 0.2% and 0.6%, mixed with N_2_O (50%) blocked the rapid recruitment of L5 neurons (fig. S12A, C). Similarly, local application of a potent and selective GABAA-R agonist muscimol induced a strong blockade of N_2_O-evoked L5 activity (fig. S12B-C). The coadministration of isoflurane (0.6%) with N_2_O (50%) also eliminated N_2_O’s antidepressant-like effect (fig. S12D). Thus, N_2_O-induced L5 recruitment is highly sensitive to acute changes in GABA receptor-mediated inhibition and supports the conjecture that N_2_O-induced L5 activity is necessary for its antidepressant-like effects.

**Fig. 5.**
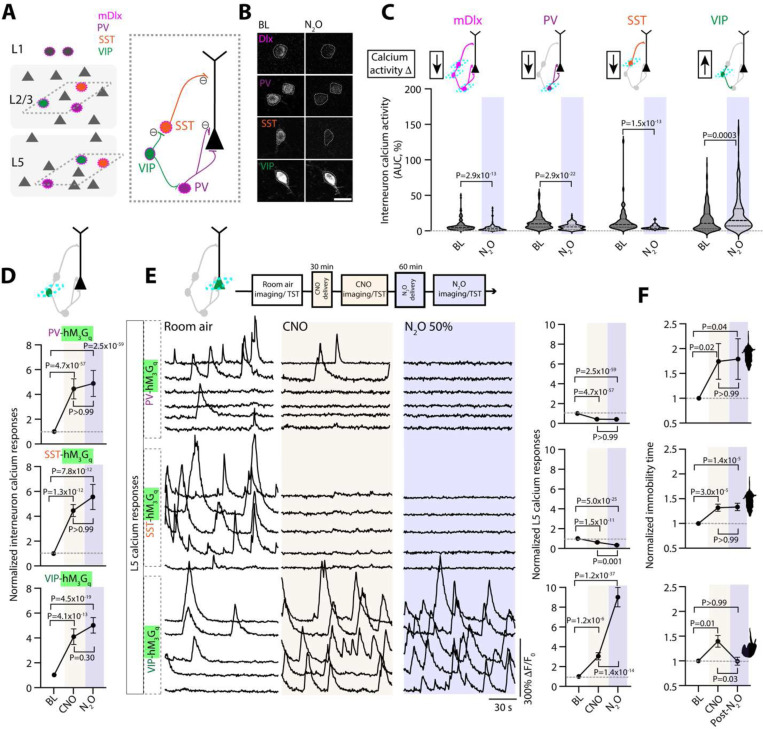
N_2_O-induced L5 activity and antidepressant-like effect requires GABAergic disinhibition. **A**, Left, Schematic of GABAergic neurons (circles) scattered amongst pyramidal neurons (triangles). While all interneurons can be labeled with GCaMP6 using the *mDlx* enhancer (outer magenta), subtypes including PV (purple), SST (orange), and VIP (green) can be specifically labeled using Cre transgenic lines depicted on right. Dendritic targeting SST cells and somatic targeting PV are inhibited (circled negative sign) by VIP interneurons. **B**, Two-photon images of peak GCaMP6 signals from interneuron subtypes under room air and N_2_O (50%). Scale bar, 20 μm. Traces in fig. S13. **C**, Summary of genetically defined interneuron calcium responses under room air and N_2_O. N_2_O induces a suppression of PV and SST spontaneous activity but activates VIP activity (Wilcoxon matched-pairs signed rank: mDlx, *n* = 133 cells from 5 mice, *P* = 2.9 × 10^−13^; PV, *n* = 145 cells from 7 mice, *P* = 2.9 × 10^−22^; SST, *n* = 63 cells from 4 mice, *P* = 1.5 × 10^−13^; VIP, *n* = 197 cells from 10 mice, *P* = 0.0003). **D**, Interneuron subtypes coexpressing GCaMP6 and DREADD-hM_3_G_q_ recorded under wakefulness, post-CNO injection, and N_2_O (50%). CNO-hM_3_G_q_ induced activation of interneuron subtypes blocked N_2_O induced suppression of PV (*n* = 84 cells from 3 mice; Kruskal-Wallis (347): *P* = 3.0 × 10^−76^ followed by Dunn’s multiple comparisons, *P* > 0.99) and SST activity (*n* = 103 cells from 3 mice; Kruskal-Wallis (68): *P* = 2.0 × 10^−15^ followed by Dunn’s multiple comparisons, *P* > 0.99). VIP cells displayed a similar trend (*n* = 97 cells from 4 mice; Kruskal-Wallis (93): *P* = 6.8 × 10^−21^ followed by Dunn’s multiple comparisons, *P* = 0.30). **E**, Left, representative GCaMP6 traces of individual L5 responses and summary of all cells (right) under room air, post-CNO injection, and N_2_O. CNO-induced activation of PV (*n* = 241 cells from 7 mice; Kruskal-Wallis (348): *P* = 3.0 × 10^−76^ followed by Dunn’s multiple comparisons, *P* > 0.99) or SST (*n* = 110 cells from 3 mice; Kruskal-Wallis (112): *P* = 3.3 × 10^−25^ followed by Dunn’s multiple comparisons, *P* = 0.001) blocked N_2_O-induced L5 activation whereas VIP promoted N_2_O-induced L5 activity (*n* = 139 cells from 3 mice, Kruskal-Wallis (169): *P* = 1.8 × 10^−37^ followed by Dunn’s multiple comparisons, *P* = 1.4 × 10^−14^). **F**, TST immobility time under the same conditions. PV (*n* = 13, Kruskal-Wallis (9): *P* = 0.01 followed by Dunn’s multiple comparisons, *P* > 0.99) and SST (*n* = 17, Kruskal-Wallis (27): *P* = 1.4 x 10^−6^ followed by Dunn’s multiple comparisons, *P* > 0.99) activation by hM_3_G_q_ blocked N_2_O-induced decrease in immobility time in CORT mice. VIP activation prior to N_2_O produced a significant decrease in immobility time (*n* = 14, Kruskal-Wallis (10): *P* = 0.006 followed by Dunn’s multiple comparisons, *P* = 0.03). Error bars show s.e.m.

While N_2_O appears to interact weakly with postsynaptic GABAergic receptors (42), the action of N_2_O on GABA-releasing interneurons is unknown. GABAergic interneurons target specific domains of pyramidal neurons and other local interneurons, providing precise control of excitatory and inhibitory outputs and cortical dynamics (Fig. 5A). A rapid shift in cortical inhibition could present one mechanism to explain N_2_O’s effect on pyramidal cell excitability. To determine if interneurons contribute to N_2_O-induced L5 activity, first we mapped spontaneous activity of interneuron responses under room air and during N_2_O exposure in mice expressing GCaMP6 under the m*Dlx* enhancer, a specific labelling strategy for GABAergic interneurons (Fig. 5B–C) (43). We found that N_2_O induced the overall suppression of interneurons activities from baseline wakefulness (110/137 cells) with only a small subset of cells becoming recruited by N_2_O (Fig. 5C, fig. S13A). Three genetically defined subtypes: PV-, SST-, VIP-expressing interneurons are subsumed within the m*Dlx*-defined interneuron population (Fig. 5A, right cartoon). We suspected that the small population of m*Dlx cells* recruited by N_2_O was specific to one of these subtypes. By taking advantage of several interneuron-specific Cre driver lines coupled with AAV transfection of Cre-dependent GCaMP6f, we examined the activity profiles of interneurons in Cg1 (Fig. 5B). Here, we found that N_2_O-induced the downregulation of PV and SST activities from baseline measurements (Fig. 5C, fig. S12A). In contrast to PV and SST activities, N_2_O increased VIP activity (Fig. 5C, fig. S12A). Therefore, N_2_O induces the rapid reconfiguration of local interneurons favoring the establishment of a disinhibitory circuit, a motif akin to ketamine (39).

To assess whether the N_2_O-induced downregulation of PV or SST activities is required for L5 activation, we attempted to counteract the suppression of interneuron activity induced by N_2_O by autonomously activating these cells using DREADD variant hM_3_D(G_q_) (fig. S13B). To drive interneuron activity *in vivo*, CNO-injected mice coexpressing GcaMP6f and hM_3_D(G_q_) specifically in PV, SST, and VIP-expressing cells induced more than two-fold increase in spontaneous calcium activity in wakefulness (Fig. 5D). CNO-mediated interneuron activation was maintained in the presence of N_2_O (Fig. 5D). Next, we activated these interneuronal subtypes individually before N_2_O while monitoring L5 neuronal activity. CNO-induced activation of either PV or SST interneurons prevented the N_2_O-induced L5 activity (Fig. 5E). Similarly, if CNO was given after N_2_O (instead of before), N_2_O-induced L5 activation was quickly abolished (fig. S13D). By contrast, CNO-induced VIP activation did not prevent the N_2_O-induced L5 activity (Fig. 5E). In control mice expressing tdTomato, CNO did not impair N_2_O’s L5 response (fig. S13C). Furthermore, the presence of N_2_O-induced L5 activation (VIP activation) led to an antidepressant-like response where its absence (PV or SST activation) did not (Fig. 5F). Taken together these experiments suggest N_2_O induces a disinhibitory circuit favoring an increase in L5 activity.

Such a dramatic shift in SST and PV-expressing populations should indiscriminately favor pyramidal cell activation (excitatory cells in L2/3 and L5) given known patterns of connectivity. While N_2_O-induced disinhibition enables L5 activity it fails to account for N_2_O’s observed L5 specificity. Therefore, we reasoned that an unidentified specific molecular interaction in both VIP and L5 neurons could explain N_2_O’s cellular and circuit changes in cortex.

## SK2 channel inhibition reproduces L5 and VIP cell activation and antidepressant-like effects

**Fig. 6.**
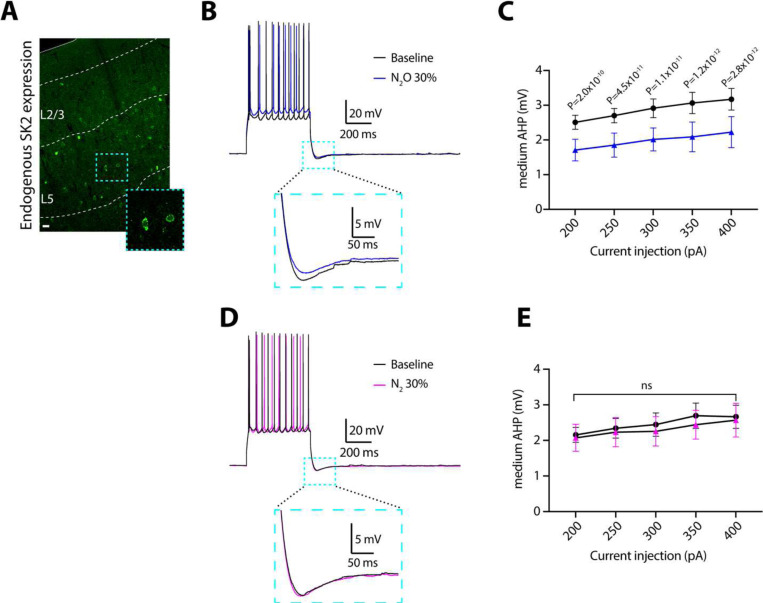
N_2_O inhibits medium afterhyperpolarization potentials in L5 neurons. **A**, Mouse Cg1 coronal section immunostained for SK2 channel. SK2-positive cells are more prominent in L5 as opposed to L2/3. Scale bar, 20 μm. **B**, Representative voltage traces of action potentials and medium afterhyperpolarization potentials (mAHPs) elicited by a 400 pA current injection under baseline conditions (black) and with 30% N_2_O (blue). Inset shows magnified mAHP as indicated by the teal box. **C**, Summary data of mAHP amplitude across different current injection intensities (200–400 pA) under baseline (black) and 30% N_2_O (blue) conditions (*n* = 9 neurons from 4 mice). Two-way, repeated-measures ANOVA shows a main effect of N_2_O on the amplitude of mAHP (F_(1,8)_ = 26.43, *P* < 0.001). Sidak’s post-hoc test results for individual current injections are shown. D, Representative control voltage traces under baseline conditions (black) and with 30% nitrogen (N_2_) (magenta). **E**, Summary data of mAHP amplitudes across different current injection intensities under baseline (black) and 30% N_2_ (blue) conditions (*n* = 7 neurons from 3 mice). Two-way ANOVA shows no effect of N_2_ on mAHP amplitude (F_(1,6)_ = 0.279, *P* = 0.616), ns: *P* = 0.616. Data are presented as mean ± SEM.

N_2_O’s well described *in vitro* NMDA-R antagonism effects are unlikely to underlie the activation of both L5 and VIP cells *in vivo* (Fig. 3). We predicted N_2_O’s ability to recruit L5 and VIP cells would rest upon a shared mechanism in both cell types, given the lack of direct connectivity between the two cell types (44), which would regulate acute changes in excitability and depolarization to drive rapid and persistent L5 activity. To this end, we explored single-cell RNA expression levels of receptors and ion channels across genetically defined pyramidal and interneuron cell types using the open source Allen Brain Cell Atlas (fig. S14A) (45). We uncovered a voltage-insensitive, calcium-sensitive potassium channel 2 (SK2 channel encoded by gene KCNN2) as a potential molecular target given: 1) its increased RNA expression in both L5 and VIP cells (fig. S14A), 2) SK2 protein immunohistochemistry and KCNN2 in situ hybridization identifying predominant L5 expression (Fig. 6A, fig. S14B) (46), 3) its role in regulating pyramidal cell intrinsic excitability and plasticity (47, 48), 4) SK channel inhibition induces antidepressant effects in rodents (49, 50).

First, we investigated the effects of N_2_O on the medium afterhyperpolarization (mAHP), the major function of SK2 channels, in Cg1 L5 neurons in acute brain slices (51, 52). mAHPs were elicited by step-current injections ranging from 200 to 400 pA. Here, bubbled N_2_O bath (30%) induced significant reduction in mAHP amplitude across all tested current injection intensities as compared to baseline measurements (Fig. 6B–C). To ensure that the observed effects were specific to N_2_O and not the result of non-specific actions of gas application, we conducted control experiments using 30% nitrogen (N_2_). As shown in Fig. 6D–E, N_2_ application did not alter mAHP amplitudes across any of the tested current intensities. These results demonstrate that N_2_O robustly reduces mAHP in cortical L5 pyramidal neurons.

**Fig. 7.**
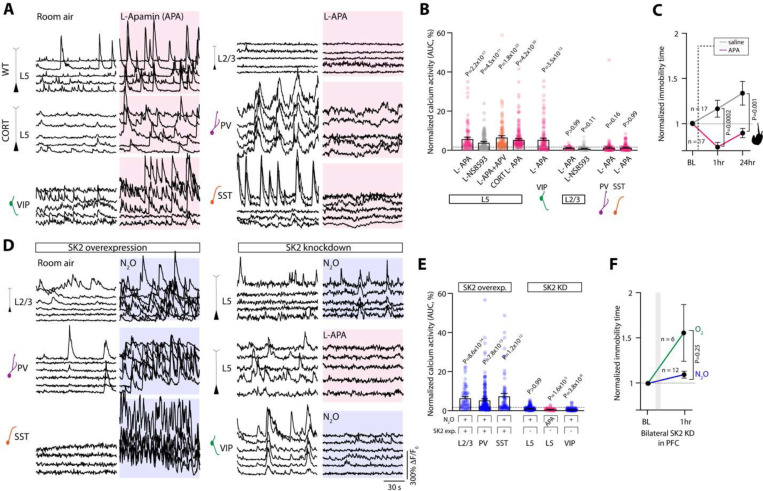
Pharmacological inhibition of SK2 channels drives rapid L5 and VIP activation and antidepressant-like response **A**, Representative GCaMP6 traces of individual L5 neurons under room air followed by local (L-) application of apamin to Cg1 (L-APA, 100 uM, 1 uL). Specific cell type noted left of traces. L5 and VIP neurons are spontaneously activated by L-APA whereas L2/3 pyramidal cells, PV, and SST cells fail to be recruited by L-APA. **B**, Summary of drug-induced inhibition of SK2 responses across different neuronal cell types (Kruskal-Wallis (455): *P* = 2.0 × 10^−92^ followed by Dunn’s multiple comparisons, L5 APA, *n* = 90 from 3 mice, *P* = 2.2 × 10^−17^; L5 NS8593, *n* = 114 from 3 mice, *P* = 4.5 × 10^−11^; L5 APA/APV, *n* = 110 from 3 mice, *P* = 1.8 × 10^−23^; CORT-treated L5 APA, *n* = 145 from 4 mice, *P* = 4.2 × 10^−20^; VIP APA, *n* = 114 from 3 mice, *P* = 3.5 × 10^−12^; L2/3 APA, *n* = 41 from 2 mice, *P* > 0.99; L2/3 NS8593, *n* = 93 from 3 mice, *P* = 0.11; PV APA, *n* =138 from 3 mice, *P* = 0.16; SST APA, *n* = 126 from 5 mice, *P* > 0.99). **C**, TST immobility time of CORT-treated mice injected with APA (0.1 mg/kg) or saline. APA-injected mice (*n* = 17) were significantly different from saline (*n* = 17) at 1 hr post injection and 24 hr later (Mann Whitney rank sum: 1 hr, *P* = 0.0002; 24 hr, *P* = 0.001). **D**, Left, individual GCaMP6 traces of L2/3, PV, SST neurons overexpressing SK2 showing effect of N_2_O (blue shaded region). Right, effect of N_2_O or APA (magenta shaded region) on L5 and VIP neurons expressing SK2-shRNA. **E**, Summary of SK2 overexpression and knockdown effects across different neuronal cell-types (Kruskal-Wallis (395): *P* = 2.3 × 10^−82^ followed by Dunn’s multiple comparisons, SK2 overexpression in L2/3, *n* = 66 from 2 mice, *P* = 8.6 × 10^−14^; PV, *n* = 228 from 5 mice, *P* = 7.8 × 10^−13^; SST, *n* = 86 from 5 mice, *P* = 1.2 × 10^−12^ versus SK2 knockdown in L5, *n* = 151 from 4 mice, *P* > 0.99; L5 APA, *n* = 81 from 2 mice, *P* = 1.6 × 10^−5^; VIP, *n* = 118 from 4 mice, *P* = 3.8 × 10^−8^). **F**, CORT-treated mice with bilateral expression of SK2-shRNA (*n* = 12) exposed to N_2_O fail to decrease their immobility time in response to N_2_O exposure as compared to O_2_ (*n* = 6; Mann Whitney rank sum: *P* = 0.25).

Next, using the approach detailed in Fig. 3, we explored whether local pharmacologic inhibition of SK2 channel function with specific inhibitor, apamin, could drive the spontaneous activation of neurons expressing SK2. Here, L5 and VIP neurons, but not L2/3 pyramidal cells or interneurons (PV or SST), were spontaneous recruited by apamin (Fig. 7A–B). NS 8593, a selective SK2 negative modulator, drove similar L5 responses (Fig. 7B). The spontaneous recruitment of L5 even occurred in the presence of local NMDA-R blockade with D-APV, confirming the NMDA-R-independent nature of this process (Fig. 7B). In contrast, local application of CyPPA, a SK2 channel activator, prior to N_2_O exposure reduced N_2_O-induced L5 activity (fig. S15A). Like N_2_O, a single apamin i.p. injection induced rapid antidepressant-like response in stressed mice (Fig. 7C).

Consistent with these pharmacologic manipulations, SK2 channel overexpression in L2/3 cell types, which normally do not express SK2 (*i.e.* pyramidal cells, PV, SST), enable acute N_2_O activation (Fig. 7D–E, fig. S15B). Knockdown of endogenous SK2 expression in L5 using Rbp4-Cre and Cre-dependent AAV encoding shRNA for KCNN2 prevented N_2_O-induced L5 activation and its ensuing rapid antidepressant-like response (Fig. 7D–F, fig. S15C-D). Collectively, these experiments suggest SK2 channel inhibition is necessary and sufficient to reproduce N_2_O’s effect on both L5 and VIP cell function and drive its antidepressant-like response.

## N_2_O-induced SK2 channel inhibition via channel pore blockade

**Fig. 8.**
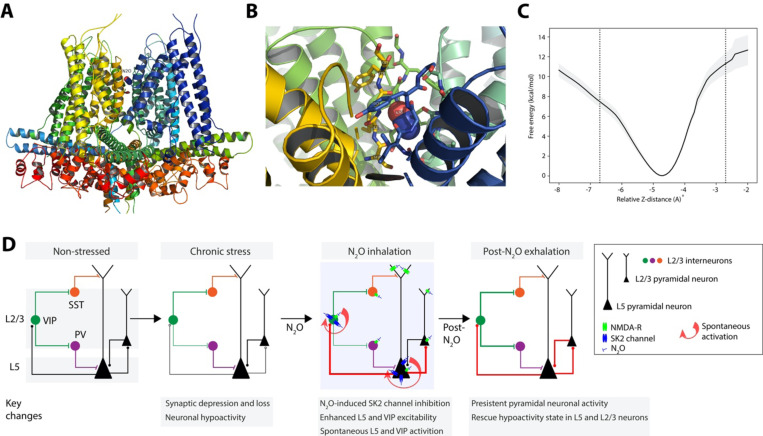
N_2_O could act as a channel pore blocker to induce SK2 channel inhibition **A**, N_2_O embedded in selectivity filter of SK2 homology model, representative snapshot from equilibrium MD simulation. **B**, Oblique view from extracellular side. **C**, PMF curve generated by ABF simulations. Free energy (y-axis) is plotted as a function of relative N_2_O z-distance (x-axis). Dotted vertical lines represent displacement of N_2_O along pore axis 2 Å in either direction, approaching the top and bottom of the filter. Shaded areas represent standard error with respect to the mean (standard deviation divided by square root of number of replicates). **D**, Working model of N_2_O-induced modulation of L5 neurons via SK2 inhibition to drive Cg1 circuit activation to rescue chronic stress-associated hypoactivity state. Chronic stress induces L5 synaptic loss (open circles) and hypoactivity (thin connections) compared to non-stress condition. N_2_O-induced SK2 inhibition in cells with high SK2 expression (L5 and VIP cells) drives their rapid spontaneous activation (circular red arrow), which can arise in the presence of NMDA-receptor blockade. L5 neurons recruited during N_2_O exposure can engage other cortical circuit elements, *i.e.* superficial L2/3 neurons upon N_2_O elimination. Following N_2_O treatment, persistent spontaneous activity in both L5 and L2/3 is likely to lead to durable changes circuit function.

We hypothesized that N_2_O might inhibit channel function by interacting with its selectivity filter, anticipating that the doses of N_2_O required for clinical effect would allow enough N_2_O molecules to bind in the filter and yield a response, even if a large energetic barrier must be traversed. To evaluate this idea, we performed all-atom molecular dynamics (MD) simulation of an SK2 homology model with a single N_2_O molecule placed manually in its selectivity filter. In equilibrium MD simulation, the N_2_O molecule remained trapped in the selectivity filter with no migration (Fig. 8A–C). To quantify the energetic barriers to N_2_O escape, we calculated a potential of mean force (PMF) profile of free energy of N_2_O diffusion along the pore axis in the region of the selectivity filter using the adaptive biasing force (ABF) method (53) which would allow recovery of a free energy profile across large energy barriers. The PMF profiles from replicate ABF simulations revealed that displacing the bound N_2_O 2 Å or more to approach either opening of the filter requires traversing an energy barrier of at least 8 kcal/mol (fig. S16). Because the SK2 structure was a homology model, we chose not to exhaustively evaluate the numerous diffusion pathways available to N_2_O moving intracellularly away from the selectivity filter (regions with larger errors in fig. S16) and therefore we draw no conclusions from these regions of the PMF. Overall, these data support the hypothesis that N_2_O may attenuate SK2 channel function in the activated state by becoming trapped in its selectivity filter (47). Moreover, SK2 with stably trapped N_2_O likely contributes to the experimentally observed persistent L5 activity following N_2_O discontinuation.

## Discussion

While N_2_O has shown therapeutic promise for severe TRD, its molecular and circuit mechanisms of action are unknown. Using *in vivo* calcium imaging across Cg1 cortical layers and cell types in mice exposed to chronic stress, we show that subhypnotic N_2_O induces rapid and specific activation of L5 neurons that persists long after N_2_O clearance from the animal. L5 activation was crucial for rescuing stress-induced circuit hypoactivity in both L5 and L2/3 and driving a rapid antidepressant-like responses. Because synaptic loss and impaired connectivity are key features of chronic stress and depressive states in rodents and humans (4, 8–10), these results provide a tangible mechanism for how a single drug treatment can re-awaken existing dysfunctional circuits without the formation of new synaptic connections and contribute to rapid changes in behaviors (Fig. 8D). The formation of synapses in response to N_2_O could contribute to stability of L5 and/or L2/3 activity and maintain these improvements over days. The extent and timescale over which N_2_O-induced activity modulates new synapse formation requires future studies.

Our studies indicate that N_2_O has important mechanisms of action in cortical circuits beyond those observed in hippocampus and other brain regions. In particular, NMDA-R inhibition does not contribute to N_2_O-induced cortical L5 activity patterns as evidenced by 1) N_2_O-induces L5 activity in presence of NMDA-R blockers or GRIN1/NR1 knockdown (Fig. 3), 2) ketamine and N_2_O differentially modulate the same population of L5 cells when the drugs given in succession (Fig. 3), 3) subcellular imaging at the level of synapses and dendritic branches reveals no upregulation of synaptic inputs or dendritic calcium spikes (Fig. 4), and 4) dendritomies of apical dendritic branches from individual cells failed to abolish N_2_O-induced L5 activity (Fig. 4). Although N₂O can recruit L5 neurons even under conditions of low NMDA receptor activity, the sustained L5 activity and neuroplasticity following N₂O elimination likely requires NMDA receptor signaling (fig. S8)(54). This is in line with recent findings suggesting that ketamine may also depend on NMDA receptor signaling to produce its antidepressant-like behavioral effects (55).

In further search of an activating mechanism, we evaluated a series of molecular targets central to L5 excitability (fig. S11-12). Enhanced GABAergic tone via inhalation of subhypnotic concentrations of isoflurane or local application of muscimol eliminated N_2_O-induced L5 activity (Fig. 12). Calcium imaging of distinct GABAergic interneuron types revealed that N_2_O engages a specific disinhibition circuit via VIP-expressing cell recruitment with downregulation of PV- and SST-expressing neuronal activities (Fig. 5, 8D). This shift in the inhibitory interneuron network favoring pyramidal cell excitation was necessary for N_2_O-induced effect on L5 activity and behavior (Fig. 5). Thus, acute modulation of GABAergic interneurons by N_2_O, like subhypnotic ketamine, yielding a disinhibited cortical circuit might represent a unifying circuit phenotype to explain how these anesthetics enact their rapid-acting antidepressant action (39, 56, 57). Dysfunctional stress-sensitive circuits could require transient drug-induced changes in inhibition to allow selective patterns of excitatory activity to propagate through cortex and engage rapid and durable forms of activity-dependent synaptic plasticity (Fig. 8D) (58).

Despite emergence of a disinhibited network, we reasoned that N_2_O-induced cell specificity must arise via an unknown molecular target, unique to L5 neurons and VIP interneurons. We identified SK2 channels as an attractive candidate with predominant L5 expression and sparse expression in L2/3. N_2_O, but not N_2_ gas, inhibited SK2-mediated mAHPs *in vitro* (Fig. 6). Local pharmacological inhibition of SK2 in Cg1 drove spontaneous activity in L5 and VIP, but not other cell types, and a N_2_O-like antidepressant response (Fig. 7). SK2 overexpression and knockdown experiments in various cell-types support the effect of N_2_O acting as a SK2 channel inhibitor. Furthermore, MD simulations advanced our biophysical theory of N_2_O acting as a blocker in the highly conserved SK2 channel selectivity filter, where N_2_O traverses a substantial energy barrier to enter or exit its selectivity filter and inhibit channel function – potassium ion efflux and hyperpolarization (Fig. 8). Altogether, these findings indicate that N_2_O-induced rapid and sustained restructuring of prefrontal L5 neuronal activity is crucial for its antidepressant action and that this effect involves novel and specific molecular actions in distinct cortical cell types.

## Supplementary Material

Supplement 1
