# Supplementary material for: Nitrous Oxide activates layer 5 prefrontal neurons via SK2 channel inhibition for antidepressant effect
Source: Res Sq. 2024 Nov 15:rs.3.rs-5141491. Preprint. [Version 1] doi: 10.21203/rs.3.rs-5141491/v1 (PMC11601843; doi:10.21203/rs.3.rs-5141491/v1)
Supplement: Supplement 1 [file NIHPPRS5141491V1-supplement-1.pdf]

Supplementary Files

This is a list of supplementary files associated with this preprint. Click to download.

- NCSupplementalDatafinal.pdf
- SDattack.mp4
- Nitrousmovie.mp4
